# Socioeconomic prospects of a seaweed bioeconomy in Sweden

**DOI:** 10.1038/s41598-020-58389-6

**Published:** 2020-01-31

**Authors:** Linus Hasselström, Jean-Baptiste Thomas, Jonas Nordström, Gunnar Cervin, Göran M. Nylund, Henrik Pavia, Fredrik Gröndahl

**Affiliations:** 1 KTH Royal Institute of Technology, Department of Sustainable Development, Environmental Science and Engineering. Teknikringen 10B, SE-133 31, Stockholm, Sweden; 2 Anthesis. Barnhusgatan 4, SE-111 23, Stockholm, Sweden; 3 University of Copenhagen, Department of Food and Resource Economics, Rolighedsvej 25, DK-1958 Frederiksberg C, Denmark and Lund University School of Economics and Management, Agrifood Economics Centre, Box 7080, SE-220 07 Lund, Sweden; 4 Department of Marine Sciences - Tjärnö, University of Gothenburg, Tjärnö Marine Laboratory, SE-452 96 Strömstad, Sweden

**Keywords:** Environmental impact, Environmental economics, Sustainability, Ocean sciences

## Abstract

Seaweed cultivation is a large industry worldwide, but production in Europe is small compared to production in Asian countries. In the EU, the motivations for seaweed farming may be seen from two perspectives; one being economic growth through biomass production and the other being the provisioning of ecosystem services such as mitigating eutrophication. In this paper, we assess the economic potential of large-scale cultivation of kelp, *Saccharina latissima*, along the Swedish west coast, including the value of externalities. The findings suggest that seaweed farming has the potential of becoming a profitable industry in Sweden. Furthermore, large-scale seaweed farming can sequester a significant share of annual anthropogenic nitrogen and phosphorus inflows to the basins of the Swedish west coast (8% of N and 60% of P). Concerning the valuation of externalities, positive values generated from sequestration of nitrogen and phosphorus are potentially counteracted by negative values from interference with recreational values. Despite the large N and P uptake, the socioeconomic value of this sequestration is only a minor share of the potential financial value from biomass production. This suggests that e.g. payment schemes for nutrient uptake based on the socioeconomic values generated is not likely to be a tipping point for the industry. Additionally, seaweed cultivation is not a cost-efficient measure in itself to remove nutrients. Policy should thus be oriented towards industry development, as the market potential of the biomass will be the driver that may unlock these bioremediation opportunities.

## Introduction

Seaweed has been called the “promising plant of the millennium”^1^ due to its comparative advantages vis-à-vis land-based biomass production. It does not need land, fresh water, fertilisers, or pest- insect- or fungicides to grow, and the biomass can be used for many purposes, such as food^2^, feed^3^, materials^4^, biofuels^5^, or as gelling or stabilising substance in a range of applications^6^. Additionally, seaweed farming provides positive externalities in terms of ecosystem services such as generating habitats for fish and crayfish species and sequestering nutrients^7^. In a recent study, it is suggested that phosphorus uptake from large-scale seaweed cultivation in China can significantly contribute to mitigating coastal eutrophication^8^, and it has also been suggested as a potential carbon sink^9,10^. Moreover, the sequestering of carbon may mitigate ocean acidification^11^.

Seaweed aquaculture predominantly takes place in Asia with China and Indonesia alone contributing 87% of the global supply, where food production and carrageenan extraction are two large industries^12^. In Europe production is currently small-scale but several drivers point towards an imminent expansion. The European Commission highlights seaweed aquaculture as having strategic potential as a contributor to blue growth by providing low-carbon and renewable products for the European bioeconomy^13^. Additionally, the Swedish Agency for Marine and Water Management identifies seaweed cultivation as a possible contributing vector for achieving Good Environmental Status with respect to eutrophication according to the Marine Strategy Framework Directive (2008/56/EC)^14^. At a global scale, seaweed cultivation has been suggested as a means by which to contribute to the reversal of key planetary boundaries transgressions^15^. Many research projects and networks are now being developed to study this industry from a range of perspectives and to unlock its potential (e.g. Seabioplas, BioMara, MAB4, EnAlgae, Seafarm, etc.). The economic profit potential is currently one of several hurdles for the development of a European seaweed industry^16^.

Seaweed farming in Europe has been subject of two major foci in the literature, beyond studies of technical issues – one being its positive environmental impacts such as provisioning of ecosystem services, nitrogen and phosphorus recovery, and possible substitution of fossil-based raw material; the other being its potential contribution to economic growth through biomass production. These different foci have implications for how seaweed farming should be understood^7^. If framed as an ‘environmental measure’, e.g. eutrophication mitigation, seaweed farming competes with other mitigating measures in terms of cost-effectiveness. If framed as an ‘industrial project’, it competes with other means of providing biomass. For industrial development in Europe, risk-factors are associated with e.g. high labour costs, yearly variation in biomass growth, the lack of mature supply chains, and permissions for allocating space. Hence, while seaweed farming may have a double dividend, it has to compete on its own within one of these two sub-regimes (environmental measure or business) unless the governing system succeeds in bridging the two by economic compensation to commercial seaweed farms such as payments for ecosystem services.

In this paper, we examine the economic potential of large-scale farming of kelp (*Saccharina latissima*) along the Swedish west coast, to (*i*) provide perspective on the potential balance between financial viability and externalities, and (*ii*) generate knowledge on potential profitability and tipping points in price and production costs. We focus on the production of seaweed for human consumption which already is a large industry in Asia. Previous studies that have assessed the profit potential for seaweed farming^16–18^ have not included the environmental impact of seaweed farming in the analysis. In this study we present a socioeconomic assessment that includes externalities from seaweed farming. In addition, we use case specific data which gives more reliable estimates of the financial flows and profitability of seaweed farming.

## Method

### General assessment method

The assessment is made in two main steps. First, a single-firm case with 2 ha (0.02 km^2^) cultivation over 10 years is studied. The cultivation site is located in the Koster archipelago. Cost data for biomass production are based on operations invoices and labour and energy cost estimations. Revenues are based on the market price for dried Seaweed (see supplementary material). The cost and revenue data are then used in a financial analysis. Externalities in terms of the most substantial positive (eutrophication mitigation) and negative (impact on recreational values) external impacts^7^ are monetized based on literature data (see Table 1 and supplementary material for details). The value of carbon sequestration is not included in the analysis, since the net effect from seaweed cultivation on the CO_2_ balance in the atmosphere is uncertain and dependent on factors such as life-length of storage in products generated by the biomass, the energy requirement in different production stages, and the possible substitution of other products for seaweed biomass^7^. Additional study on the associated carbon cycles is needed to estimate these values in monetary terms. Financial and external net values are then combined to provide an overview of the overall economic balance. This methodology is in line with cost-benefit analysis^19^.

**Table 1:** Summary table for the variables used in the analysis.

Variable	Unit	Midpoint value	Worst	Best	Reference
	***Production/output biomass***				
Long line	Km per hectare	2.34			Case data.
Production: wet weight	Tons per km long line/year	8	7.5	15	Case data.
Dried seaweed as share of wet weight		0.1789	0.1737	0.1842	Based on 18.5% moisture content in dried product, midpoint of a 15–22% interval, where 15% is considered a conservative lower end of interval and 22% is max recommended^36^.
Dry weight share of wet weight (i.e. no water at all left)		0.151			^37^
Production: dried seaweed	Tons per hectare	3.3497	3.1403	6.2806	Calculations from above.
	***Financial costs and benefits***				
Material every year	EUR per 2 ha	31 657	32 126	31 187	Case data.
every 5^th^ year	EUR per 2 ha	7 192	7 192	7 192	Case data.
every 10^th^ year	EUR per 2 ha	46 731	54 240	39 223	Case data.
Labour every year	EUR per 2 ha	54 451	61 653	47 249	Case data.
every 10^th^ year	EUR per 2 ha	6 695	8 123	5 268	Case data.
Energy every year	EUR per 2 ha	1 089	2 119	58	Case data.
Sales value (dried seaweed)	EUR per kg dried seaweed	31	10	52	Conservative estimate based on^38–40^, and current market price in Sweden. See supplementary material for details.
Productivity growth		2.4%			See supplementary material for details.
		***Externalities***			
N content	Kg per ton dwt	16			^5^
Economic value of N	EUR per kg N	7.6	3.6	11.5	^24,28–31^
P content, kilo/ton dry weight	Kg per ton dwt	2.4			^5^
Economic value of P	EUR per kg P	86.5	0	172.9	^28,29^
Total recreational values west coast (“Consumer Surplus”)	Thousand EUR	1 805 800			Calculations based on^24^ and^25^
Share of Consumer Surplus loss at max potential scale		6%	10%	2%	Assumption.
		***Discount rate***			
Discount rate		4%	6%	2%	Assumption.^41^

Second, the analysis is repeated in a scenario depicting rapid scaling-up of the single firm case on the Swedish West Coast over 40 years. The scenario itself is back-cast from a maximum cultivated area of 338 km^2^ achieved at year 40, identified as suitable for the cultivation of *Saccharina latissima* on the Swedish West Coast^20^. This scenario would require 28% growth in cultivation area annually, which is significantly higher than the current annual growth of farmed aquatic plants globally (around 8% average growth between 1990 and 2016; with the highest growth in Indonesia around 20%)^12,21^.

A project is profitable if benefits exceed costs on a societal level^19^. Technically, this is the case if the net present value (NPV; Eq. 1) is greater than zero. This implies an aggregation of costs (C) and benefits (B) over a given time period (T). Future costs and benefits are discounted using a discount rate (r).

1 $$NPV=\mathop{\sum }\limits_{t=0}^{T}\frac{1}{{(1+r)}^{t}}({B}_{t}-{C}_{t})$$

where t denotes the time when the respective cost or benefit item occurs.

### Indata: financial flows and ecosystem services

Table 1 shows the estimated values for the variables included in the analysis. Details on the assumptions can be found in the supplementary material. Worst and best values are presented for all variables, except for when an exact point estimate has been provided. In the following analysis, the midpoint values have been used for all variables. In the sensitivity analysis we use the worst and best values for each variable to evaluate the robustness of our results.

## Results

**Figure 1:**
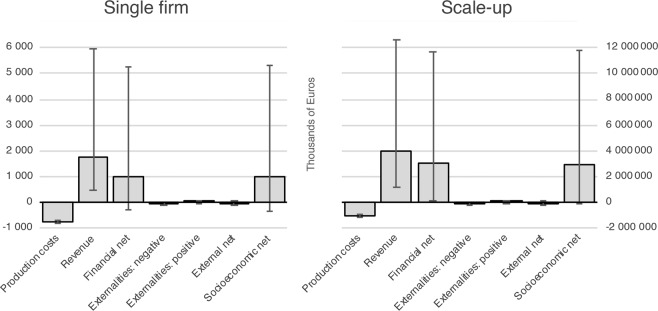
Net present values for single-firm 2 ha scenario and scale-up scenario where 338 km^2^ is used for seaweed cultivation (thousands of Euros). Error bars represent results when all variables are simultaneously at their worst case vis-à-vis best case values according to assumptions in Table 1.

We begin our analysis by studying the single-firm case to see what drives the results in the short run. We then turn to the scale-up scenario where 338 km^2^ (the max potential^20^) are used for seaweed cultivation. The left chart in Fig. 1 shows the results based on a single firm cultivating two hectares over ten years. The values are expressed as net present value (NPV) in thousands of euros. As Fig. 1 shows, both the financial NPV (€1 018 thousand) and socioeconomic NPV (€979 thousand) are positive. The variables having the largest impact on both the financial and socioeconomic NPV are the production costs (€734 thousand) and revenues (€1 752 thousand), while the externalities have a minor impact. The positive externalities, N and P uptake, amount to €6 thousand and €10 thousand respectively, while the negative externality (loss of recreation possibilities) amounts to minus €54 thousand. This suggests that the values of negative externalities cancel the positive ones out, resulting in an externality NPV estimated to minus €38 thousand. The break-even sales price in the single-firm case is 13 €/kg dried seaweed. This is well below the midpoint sales value and close to the lowest predicted sales value (see Table 1). The right chart in Fig. 1 shows the results for the scale-up scenario where the total area of 338 km^2^ is used for seaweed cultivation. The figure shows the same pattern as for the single-firm case, with large financial and socioeconomic NPV’s (€2.9 billions).

The error bars in Fig. 1 represent extreme scenarios in which all input parameters in the economic model are set at their best and worst values, respectively. Such scenarios are unlikely. The error bars however show that the likelihood of a negative net present value given the parameter ranges specified in the model are minimal. As the figure shows the single most important variables driving uncertainty in this model are the assumptions that define revenue.

The scale-up scenario implies production during year 40 of approximately 1 000 000 tons of biomass (wet weight). Such volume still remains a rather small share (3%) relative to current global production of farmed aquatic plants^12^. Concerning N and P uptake, this level of production in year 40 would sequester 1 529 tons N and 229 tons P. The yearly anthropogenic net load to these basins is 18 400 tons of N and 380 tons of P in year 2014^22^, which implies that this cultivation size would enable sequestration of 8% (N) and 60% (P) of the annual anthropogenic net load to these basins.

**Figure 2:**
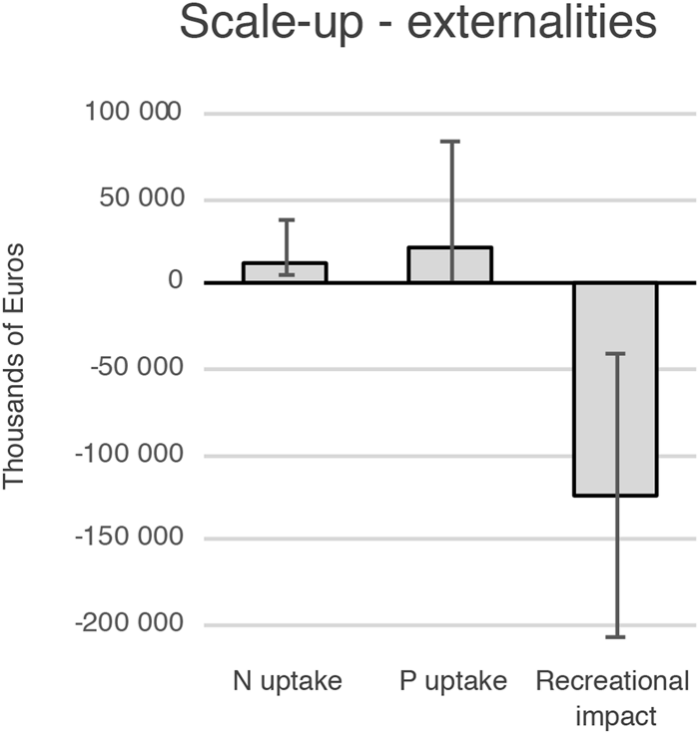
Net present values of externalities for scale-up scenario where 338 km^2^ is used for seaweed cultivation (thousands of Euros). Error bars represent results when all variables are simultaneously at their worst case vis-à-vis best case values according to assumptions in Table 1.

At this level of seaweed cultivation, the NPV of the N and P uptake amounts to €13 million and €23 million respectively (Fig. 2). The recreational impact is estimated to minus €124 million, resulting in a negative socioeconomic NPV of €88 million, considering only the externalities. Compared to the revenues the N and P values amounts to 0.3 and 0.6% respectively, while the negative externality amounts to 3.1%. As the production costs are higher than the value of reduced eutrophication, this suggest that seaweed cultivation is not an economically profitable measure for eutrophication mitigation *per se*.

Additionally, the studied setup is not a cost-effective measure for reducing N and P, if one disregards sales revenue. Marginal costs are 800 €/kg N and 5 400 €/kg P, which is higher than the marginal cost of most land-based measures^23^. However, taking revenue into account, the marginal cost for N and P reductions using seaweed cultivation is negative, making this measure highly cost-effective.

An additional robustness check of the results was done by changing the midpoint value of the variables to either the worst or best value presented in Table 1. The analysis is done for each variable separately. For both the single-firm and the scale-up scenario the results are most sensitive to changes in the sales value. If the max value (€52/kg) is used the socioeconomic NPV increases with 121% to €2 166 thousand for the single-firm scenario and with 94% to €5 598 million for the scaled-up scenario. Applying the min value (€10/kg), the socioeconomic NPV fall to €−208 thousand and to €170 million for the single-firm and scale-up scenario, respectively.

For the single-firm scenario the variable that has the second largest impact on the socioeconomic NPV is the production of seaweed per km long line per year, whereas the financial discount rate has the second largest impact in the scale-up scenario. Varying the economic value of N and P uptake has the smallest impact on the result. More in-depth sensitivity analysis is presented in the supplementary material.

## Discussion and Conclusions

Our results show that seaweed cultivation has the potential to become a highly profitable industry in Sweden and that the monetary values of externalities are rather small compared to the financial values generated. Values forgone due to interference with recreation may however be substantial. Large-scale seaweed cultivation along the Swedish west coast is also an imaginable tool in future eutrophication combating. Our analysis suggests that large-scale seaweed cultivation may sequester 8 percent of annual anthropogenic net nitrogen and 60 percent of annual anthropogenic net phosphorus inflows to the basins on the Swedish West coast.

Although the sensitivity analysis suggests that our results are robust, our data have some limitations. The largest uncertainty beyond what the analysis is able to capture is arguably for the externalities. Due to lack of case-specific studies for recreational loss due to seaweed cultivation, a share of the consumer surplus for recreation (as estimated using previous studies^24,25^, see supplementary material) has been applied to measure the loss. The true value can be both larger and smaller than the values we have used, and the extent to which recreational values are lost depends on locations, seasons and design of the cultivation sites. Given that seaweed is harvested in May and a new cultivation cycle begins in the fall, it is possible that recreational losses here have been overestimated since the main recreational season in Sweden is during the summer months. A recent study among residents along the Swedish west coast indicates e.g. that two out of five respondents are not concerned that cultivation sites will have an impact on leisure boating^26^. In addition, increasing activity along the coast could also bring cultural values and food tourism.

The costs presented in this study are estimated from inventories of material and energy use and estimations of labour requirements per hour, based on an actual cultivation setup in Sweden^27^. In terms of transferability of these cost estimates to other setups or locations, we acknowledge that some costs may have been circumvented owing to local conditions, for instance, in Sweden there are no specific licensing fees. Relative to other economic assessments in literature^16–18^, there are specific differences in terms of inventory items, costs and estimated lifetime of investments; however, on the whole, costs are comparable.

The economic values of N and P uptake are based on previous valuation studies on marine eutrophication^24,28–31^. Although the assessment shows that substantial amounts of these nutrients can be sequestered, the value of this sequestration is still low compared to the financial turnover. This pattern does not mean that the socioeconomic value of eutrophication mitigation is small; it could also be interpreted that potential revenue streams in a future seaweed industry are large. However, undeniably, there is uncertainty in these estimates in terms of how well they capture the values at risk from marine eutrophication. An additional aspect to consider concerning the bioremediation activity of a future seaweed industry is phosphorus as a resource. As phosphorus becomes scarcer^32,33^, recovering it from cultivated seaweed biomass could open up new opportunities and may increase the socioeconomic value of a seaweed industry.

While eutrophication mitigation and recreational impacts are identified as the most significant externalities^7^, there are other positive and negative externalities as well, not being monetized in this study; positive effects such as habitat generation for fish and crayfish, reduction of ocean acidification, carbon sequestration and possibly negative effects from e.g. shading effects on bottom fauna. Concerning other externalities, these are likely to be dependent on the specific case setup and location choice. Further study is required to conclude on the magnitude of these externalities in monetary terms.

Given the findings here presented, it is clear that policy instruments such as subsidies for nutrient uptake, based on the economic value of nutrient sequestration, will not be a tipping point for market development. Additionally, if the profit potential on the market for the biomass will not be realized, bioremediation through seaweed cultivation is not a cost-effective measure. The potential of the bioeconomy is instead in market development, where a mature market would lead to significant ancillary benefits in terms of providing a renewable resource and mitigating marine eutrophication. In contrast to the traditional view in environmental economics on market failures needing ‘corrections’ in terms of policy instruments, this analysis shows a new promise for the role of the market in a transition to a sustainable future.

**Figure 3:**
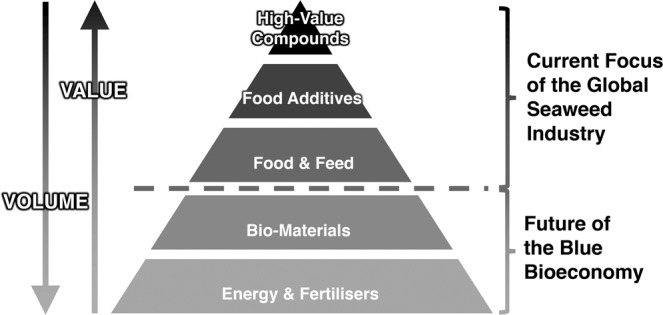
Value pyramid for the seaweed industry. Source:^34^, adapted from^35^.

Consider the classic biomass value pyramid applied to seaweed biomass (see Fig. 3): the current global seaweed industry primarily produces seaweed as food for human consumption and some higher-value/lower-volume products, however, lower-value/higher-volume products such as seaweed-based biomaterials, bioenergy and fertilisers are not yet produced on a significant scale. Ultimately, the prospects of cultivated seaweed biomass to contribute to more sustainable futures will largely depend on the bottom lines of seaweed cultivation and downstream processing, and accordingly, the viability of replacing fossil-based products. Our model indicates that cultivated seaweed biomass sold for food can indeed be profitable, even comfortably profitable in a Scandinavian context. The biomass could possibly also be produced for lower-value/higher quantity products, and with continuing policy efforts to steer away from finite resources, the market for such products may develop into generating higher returns for producers. Given the urgent need to phase out fossil-based energy, materials and fertilisers, seaweed biomass may well be among the key bio-resources for the next decades.

## Supplementary information

Supplementary information Hasselström et al.
