# Supplementary material for: Socioeconomic prospects of a seaweed bioeconomy in Sweden
Source: Sci Rep. 2020 Jan 31;10:1610. doi: 10.1038/s41598-020-58389-6 (PMC6994625; doi:10.1038/s41598-020-58389-6)
Supplement: Supplementary file 1 — Supplementary information Hasselström et al. [file 41598_2020_58389_MOESM1_ESM.pdf]

Supplementary material

Socioeconomic prospects of a seaweed bioeconomy in Sweden

Linus Hasselström[1,2]
Jean-Baptiste Thomas[1]
Jonas Nordström[3]
Gunnar Cervin[4]
Göran M. Nylund[4]
Henrik Pavia[4]
Fredrik Gröndahl[1]

[1] KTH Royal Institute of Technology, Department of sustainable development, environmental science and engineering. Teknikringen 10B, SE-133 31 Stockholm, Sweden.

[2] Anthesis. Barnhusgatan 4, 111 23 Stockholm.

[3] University of Copenhagen, Department of Food and Resource Economics, Rolighedsvej 25, DK-1958 Frederiksberg C, Denmark and Lund University School of Economics and Management, Agrifood Economics Centre, Box 7080, SE-220 07 Lund, Sweden

[4] Department of Marine Sciences - Tjärnö, University of Gothenburg, Tjärnö Marine Laboratory, SE-452 96 Strömstad, Sweden

1. Production costs and revenue

The costs presented in this study are estimated from inventories of material and energy use and estimations of labour requirements per hour, based on knowledge developed in the Seafarm [Formas 2013-92] and Mistra AquaAgri Kelp projects, which together have led to a start-up company called KosterAlg AB. As proof of concept research projects with the intent of laying the foundations for a subsequent industry on the Swedish West Coast, cultivation operations were based at and used the facilities at the Tjärnö Marine Laboratory, Sweden, thus meaning that premises and basic laboratory equipment were available at a relatively affordable rate, and as such, some costs may not be representative of equivalent costs as might be experienced by a company that would not have access to such facilities. To handle this, such costs were estimated from quotes obtained from local companies, for instance, for the rental of a hatchery facility for three months of the year and for a small office space and storage space for the company year-round. Other costs may also have been circumvented owing to local conditions, for instance, in Sweden there are no licensing fees for seaweed cultivations. Relative to other economic assessments in literature [1-3], there are specific differences in terms of inventory items, costs and estimated lifetime of investments; however, on the whole, costs are comparable.

Table 1. Overview of production costs.

	Investment frequency	MATERIAL		ENERGY			LABOUR		
		Euros	Certainty	kW	Euros	Certainty	hrs	Euros	Certainty
1. Spore preparation	Yearly	3673	Case	326	21	Case	16	591	30%
	5th year								
	10th year	783	Med/High	< 100					
2. Seeding	Yearly	3866	Case	560	37	Case	63	2328	20-50%
	5th year	104	Case						
	10th year	3518	Case	< 100			12	443	20%
3. Cultivation	Yearly	16193	Case	< 100			158	3961	20-30%
	5th year	7088	Case						
	10th year	31733	Case	< 100			197	4824	20-50%
4. Harvest	Yearly	5467	Case	< 100			78	1943	50%
	5th year								
	10th year	3189	Case	< 100					
5a. Drying (KosterAlg case data from 2018)	Yearly	2928	Case	31525	2062	Case	689	14403	50%
	5th year								
	10th year	15017	Case	< 100			112	2855	20%
5b. Drying (Outsourced)[1]	Yearly	1989	Reference	Included in material cost estimation			Included in material cost estimation		
	5th year								
	10th year								
6. Maintenance	Yearly						110	38426	10-50%
	5th year								
	10th year								

TOTAL COSTS									
Lower-bound estimate	Yearly	31187	Euros		58	Euros		47249	Euros
	5th year	7192	Euros						
	10th year	39223	Euros					5268	Euros
Upper-bound estimate	Yearly	32126	Euros		2119	Euros		61653	Euros
	5th year	7192	Euros						
	10th year	54240	Euros					8123	Euros
Midpoint	Yearly	31657	Euros		1089	Euros		54451	Euros
	5th year	7192	Euros						
	10th year	46731	Euros					6695	Euros

Spore preparation. MATERIAL: Premises, laboratory equipment, filtration system, lighting system. ENERGY: Filtration system, lighting system, sourcing of seawater (pump), aeration, temperature control. LABOUR: estimated 16 hours (high wage labour) per year which includes collection of parent specimen, monitoring of tumble culture and other lab procedures [4].

Seeding. MATERIAL: Premises, laboratory equipment, hatchery tanks, filtration system, lighting system, collectors, seeding line and consumables. ENERGY: Filtration system, lighting system, sourcing of seawater (pump), aeration, temperature control. LABOUR: estimated 63 hours (high wage labour) per year for lab procedures including preparing the collectors, preparing and weekly change of the culture medium, setting up apparatus, monitoring, and 12 hours (high wage labour) per ten years for the initial set up of the hatchery [4].

Cultivation. MATERIAL: Premises for logistics and equipment storage, transport and installation with Nereus (24hrs/10years), monitoring with the dinghy (12hrs/year), anchors, buoys, ropes (longlines, mooring lines, lateral anchors and buoy-to-longline ropes), chains, shackles. ENERGY: Negligible contributions to (electrical) energy use; boat fuel included in material costs. LABOUR: 158 hours (mixed wage labour) per year for logistics, installation at sea, seeded line deployment and monitoring, and 197 hours (mixed wage labour) per ten years for the initial logistics, prepping material onshore and deployment of the infrastructure at sea.

Harvest. MATERIAL: Harvesting with Algot1 (26hrs/year), harvesting tanks and transport vessel (26hrs/year). ENERGY: Negligible contributions to (electrical) energy use; boat fuel included in material costs. LABOUR: 78 hours (mixed wage labour) per year for the harvest.

Drying Koster Alg 2018. MATERIAL: Premises, outdoor drying polytunnel, drying equipment, water activity and water content meters, packaging, and biomass transport onshore. ENERGY: Measured energy consumption 31525kWh priced at 0.06 Euro/kWh. LABOUR: estimated 689 hours (low wage labour) per year for onshore handling and drying, and 112 hours (mixed wage labour) per ten years for the initial set up of the drying facility.

Drying BIM. Estimated as a total cost [1].

Maintenance. LABOUR: estimated at 108 hours (mixed wage labour) per year, covering maintenance activities such as diving inspections to check the infrastructure at sea, hatchery facility and drying facility maintenance, and the cleaning of longlines and other equipment after the harvest. Material and energy costs for maintenance activities are included in yearly and 5-year costs. Furthermore, herein is also included the cost of hiring an employee at 50 % for year-round administration, marketing, logistics, and to help out with various aspects of the business, maintenance and operations including in the hatchery (spore preparation, seeding), cultivation, harvest, drying.

Energy only includes electrical energy processes that consume a total approximately greater than 100kW. Fuel and other energy forms are included in material costs.

Certainty refers to how certain this information is and/or by what degree it is likely to vary in the present case. Certainties are presented averages or ranges from certainty data collected during interviews. The words or numbers used pertain to suggesting how certain this information is and/or by what degree it is likely to vary in the present case.

Exchange rate used is 0,1038 EUR/SEK based on average exchange rates for 2017 [5].

Energy costs based on price per kWh for a medium size industry client (category IC, 500 – 2000 MWh per year), average price 2012-2017 [6]. Prices include fixed fees and environmental taxes.

Material costs

The supply chain in the present calculations begins with the hatchery. The hatchery involves a series of laboratory-based steps that result in the provision of dozens of coils of string (hereafter collectors), covered in juvenile macroalgae ready for deployment to Sea. The premises used for the Seafarm project are located at the Tjärnö Marine Laboratory. These premises are owned by Akademiska Hus and rented at very modest rates, however, in the present study it is assumed that a fully equipped hatchery facility would be rented at a cost of approximately 70 euros per day, for a period of three months during which hatchery operations take place. The key requirements of a hatchery facility are space, temperature control and availability of seawater. Material costs of the spore preparation and seeding processes include aquaria, grow lights, the seeding line and aforementioned collectors, growth medium (including nutrients and ultra-filtered seawater pumped from the sea), and other basic laboratory equipment (e.g. flasks, buckets, tissue paper, etc). The costs are presented separately for each process (spore preparation and seeding/juvenile nursing) and reflect the total required to supply the 2 ha cultivation located in the Koster archipelago, equivalent to a little less than 5 km of longline.

The cultivation step includes costs for the year-round rental of a small office and storage space for logistics and operations, the cultivation infrastructure itself, transportation to and from the cultivation site, material maintenance and operation of processes throughout seaweed maturation, including regular monitoring of the biomass and infrastructure. The cultivation infrastructure consists of 26 longlines (190 m each) covering 2 ha in the Kosterhavets National Park. The longlines feature a lateral anchoring line traversing the mid-point of each longline in order to secure the relatively extended longline configuration (190 m rather than conventional 100 m longlines), chains and a shackle connecting each 600kg concrete anchor to large marker buoys at the end of each longline and directly above the anchors, and small buoys every 10 m to keep the longline at a uniform depth of approximately 2 m.

Transportation of material, biomass and labour to and from the cultivation site is done with three different vessels, and the harvest itself facilitated using a motorised barge. The first vessel, Nereus, is a research vessel based at Tjärnö and is used for the installation of the infrastructure and some maintenance operations. The second vessel is a small dinghy and is used to monitor the biomass or check the infrastructure after heavy weather. The use of Nereus costs approximately 415 euros (4000 SEK) per hour and includes a skipper, while the dinghy costs approximately 104 euros (1000 SEK) per hour. The third vessel is a privately-owned vessel, hired for 104 euros (1000 SEK) per hour to transport the harvested biomass from the cultivation back to shore. Stated costs also include fuel. The motorised barge used to facilitate the harvest, Algot1, was custom built for the task and it is estimated that over an anticipated 30-year life expectancy, it's cost averages out to approximately 104 euros (1000 SEK) per hour. The harvest includes all the harvest of the biomass from the infrastructure, packing it for transport and offloading to a dock. Finally, and as aforementioned, the costs for biomass drying were particularly uncertain given that these methods are still being tested, developed, and are far from optimised. To handle this uncertainty, a higher and lower bound estimate were separately calculated. The higher bound estimates are based on KosterAlg's

unoptimized summer 2018 harvest practices, and include the facility, drying equipment (agricultural polytunnel, structure to hang longlines on, and drying equipment for final dewatering), and packaging. The lower bound costs are estimated using the mean (60 euros per two tonnes wet weight) of the cost range (45 to 75 euros per two tonnes wet weight) suggested by Watson and Dring [1], the range itself based on an Irish case study which described a unit specifically designed and built for the drying of seaweed [7]. A midpoint estimate from these upper and lower bound extremes is used in the main model. To evaluate how sensitive the results are for changes in the cost of drying we doubled the cost in a sensitivity analysis. The results show a minor effect on the social net present value (SNPV) of -0.7 and -0.3% for the single-firm and scale-up scenario, respectively.

Due to differences in the life expectancy of different inputs of the system or due to differences in the frequency of procedures, costs are categorised into initial set-up (once every ten years), some particular maintenance costs (every five years) and operational costs (yearly) for each of the aforementioned steps. The model further assumes that none of the infrastructure or components of the system will last more than ten years, that all components will be replaced on the tenth year at the latest.

Given the small scale of the Seafarm project's operation, nearly all of the material inputs appearing in the invoices were ordered in relatively small numbers; large bulk order discounts did not typically apply nor do other economies of scale that could be expected. Furthermore, this project serves to produce biomass for research purposes – it is neither cost efficient, nor optimised as a business. It is anticipated that cost savings would be possible relative to the cost data collected for this study. Finally, the authors recognise that the costs included in the model may not be comprehensive, that some costs incurred may have been left out of the model as aforementioned, notably regarding laboratory equipment, premises or licensing costs. We therefore carry out a sensitivity analysis to evaluate how sensitive our results are to increases in the cost of spore preparation and seeding, for which laboratory equipment and premises are important costs. If the cost for spore preparation is doubled the SNPV decreases with 3.0 and 1.2% for the single-firm and the scale up-scenario, respectively. If the both the cost for spore preparation and seeding is doubled, the SNPV decreases with 6.3 and 2.9% for the single-firm and the scale up-scenario.

Energy-use

Energy-use and costs are based on and/or modelled from case data and the practices undertaken within the Seafarm project since 2014. Energy-use pertains exclusively to the use of electrical energy. Fuel costs, e.g. for the boats, are excluded from the energy-use estimations, however they are included in the material costs. Where direct measurements of (electrical) energy use were not available, these were modelled using specifications of machinery or equipment. When energy use was measured or estimated in models to be less than 100 kW per year, those specific uses were considered negligible and were excluded from the present study. The energy use of set-up and maintenance activities aggregated to less than 100 kW, and thus are excluded from the following table.

Costs for energy are based on Price per kWh for a medium size industry client (category IC, 500 – 2000 MWh per year), average price 2012-2017. Prices include fixed fees and environmental taxes. VAT not included [6].

Labour costs
Labour costs are presented separately from those of the supply chain. The model accounts for two types of labour cost: *low wage* and *high wage*.

Low wage
Hourly wage assumed to 15.3 €/hour (147 SEK/hour), which represents average wage for blue-collar workers in fishery, agriculture and forestry in Sweden 2016 [8].

Social fees: 37 % (32 % social fees, 5 % insurance fees) [9]. Calculation based on monthly wage, recalculated by multiplying hourly wage with 168.7 labour hours per month during 2016.

Total hourly cost: 20.9 €/hour (201 SEK/hour [201.4]).

High wage
Hourly wage assumed to 26.3 €/hour (253 SEK/hour [252.5]), which represents average wage for high-educated employees (>3 years higher education) in the private sector in Sweden 2016 [10] (recalculated from 4422 €/month (42 600 SEK/month) based on 168.7 labour hours per month average for 2016).

Social fees: 41 % (32 % social fees, 5 % insurance fees for monthly salaries below € 3847 (SEK 37 063), 31 % insurance fees for monthly salaries above € 3847 (SEK 37 063)) [9]. Calculation based on monthly wage, recalculated by multiplying hourly wage with 168.7 labour hours per month during 2016.

Total hourly cost: 37 €/hour (356 SEK/hour [356.1]).

The number of high or low wage labourers employed and the man-hours required to complete specific tasks of the supply chain were estimated based on the practices performed at the Seafarm and AquaAgri Kelp project since 2014 for the hatchery, cultivation, harvest and maintenance operations. Labour requirements for the drying were based on the practices at KosterAlg in the summer of 2018.

Unlike the energy and material maintenance costs, labour costs are estimated separately from each of the steps of the supply chain and expressed per year. These include the labour hours for maintenance tasks in the hatchery, to conduct diving inspections and replace parts of the infrastructure at sea, for cleaning and preparing longlines and buoys for the following year following the harvest, and to prepare and close the drying facility for each harvest season.

In addition to the specific operational labour hours accounted for in the model, this study further assumes that the business would hire one employee at 50 %, which would cost an estimated 35200 euros per year, including all taxes, fees and benefits.

Revenue data
Price for seaweed is based on dried seaweed to the food market. Le Bras, et al. [11] suggests a price ranging from 10 €/kg for seaweed pasta to 150 €/kg for nori. Tasende and Peteiro [12]

estimate 40-49 €/kg for Kombu. Organic Monitor [13] finds that price for Kombu varies between 49-163 €/kg depending on supplier. KosterAlg AB in Sweden sells dried cultivated kelp for food at 52 €/kg (500 SEK/kg) [14]. Here, we assume 10-52 €/kg with the midpoint estimate 31 €/kg.

Productivity growth

Productivity growth, defined as relative increase in added value per labour hour, leads to a lowering of labour-related production costs over time, i.e. the same output can be produced using fewer labour hours.

Given the early life of seaweed cultivation in Sweden, a high productivity growth due to learning and new technology is assumed. Here, we assume the productivity growth to be similar to that of the manufacturing sector which is the sector with the highest long-term productivity growth in Sweden; 3.9 % between 1981 and 2015 [15]. For comparison, the productivity growth in the private sector totally in Sweden was 2.2 % between 1981 and 2015 [15].

A part of this productivity growth is reflected in real wage increases over time. Real wage increase in Sweden was 1.5 % between 1981 and 2015 [16].

Hence, the wage-adjusted productivity growth, that are used in our calculations, is estimated as the difference between the productivity growth (3.9 %) and the real wage increase (1.5 %): 2.4 %.

2. External costs and benefits

Nitrogen and phosphorus uptake

Content of N and P has been estimated to, respectively, 16 and 2.4 kg per ton dry weight biomass [17]. The monetization of this nutrient uptake is based on existing values for eutrophication mitigation from the literature. A number of different willingness to pay estimates from individual WTP-studies or meta analyses in Sweden are available, representing the societal value of eutrophication mitigation from reductions of N and P (respectively) in marine waters. For N, an interval of 3.64 – 11.48 thousand EUR per ton N is used [18-22]. Ahlroth [18], Kinell, et al. [19] and Noring [20] conducted meta analyses of a number of WTP studies. Czajkowski, et al. [21] conducted a travel cost study where recreational impacts of an improved environmental status is assessed for the Baltic Sea including the Swedish west coast. Ahtiainen, et al. [22] conducted a willingness to pay study of a scenario in which the Baltic Sea Action Plan [23] with regards to eutrophication is reached. Swedish estimates from these latter two studies are divided by the BSAP nitrogen reduction requirements for Sweden, as recommended in Söderqvist, et al. [24] to obtain a per unit nitrogen value. It should be noted that these estimates are developed for the Baltic Sea and Kattegat and not the adjacent Skagerrak, which is less eutrophicated. Hence, an interval including the lower estimates from e.g. Kinell, et al. [19] is motivated.

For P, an interval of 0 – 172.78 thousand EUR per ton P is used [18] assuming that the value could be as low as zero given that the P reduction quota for Skagerrak according to the Baltic Sea Action Plan is already fulfilled [25].

Recreational values

Concerning recreational values forgone due to space conflicts, we assume that a share of the recreational values measured as consumer surplus (CS) for marine recreation along the west coast is forgone if seaweed cultivation is developed. The total CS for marine recreation in Sweden has been estimated to approximately 4.6 billion EUR, or approximately 98 EUR per trip using the travel cost method [21]. To translate this to the Swedish west coast we use data on coastal recreation [26], which indicates that the share of recreational trips to the west coast is approximately 38 % of total for Sweden, or approximately 2.45 trips per Swede and year while the number of trips per Swede anywhere to the Swedish coast is 6.42 per year. This results in a CS for west coast recreation estimated to 1756,69 mEUR per year. We assume that for each year there is an increased loss of recreational values proportional to the cultivation area, to reach 2-10 % loss of CS in the max potential scenario with a midpoint of 6%.

These estimates account for values forgone due to space conflicts. Recreational benefits could also occur due to water quality impacts of N & P removal. These benefits are however captured in the estimates for value of N & P uptake, as specified above.

Discount rate

Evans [27] argues for a social discount rate of 3-4 percent for the more developed EU countries. At present the Swedish Transport Administration uses a social discount rate of 3.5 percent for investment in roads and railways [28]. In the analysis, we use a baseline (midpoint) discount rate of 4% for both the social and financial analysis and have conducted sensitivity checks using min and max values of 2% and 6%, respectively.

Sensitivity analysis

A robustness check of the results was done by changing the midpoint value of the variables to either the worst or best value presented in Table 1. The analysis is done for each variable separately.

For both the single-firm and the scale-up scenario the results are most sensitive to changes in the sales value. If the worst value 10 €/kg dried seaweed is used, the SNPV for the single-firm scenario falls to €-208 thousand and for the scale-up scenario to €170 million. The reason that the scale-up scenario becomes positive is due to the productivity growth. Applying the best value 52 €/kg dried seaweed increases the SNPV to €2166 thousand (or with 121%) for the single-firm scenario and to €5598 million (94%) for the scaled-up scenario.

For the single-firm scenario the variable that has the second largest impact is the *production of seaweed* per km long line per year, followed by *financial discount rate, recurrent and construction costs*, *recreational loss* and the *value of N and P uptake*. Changing these variables varies the SNPV with ± 11.0 for the most sensitive variables to ± 1.3% for the least sensitive. Since we use a conservative value, close to the min/worst value, for production of seaweed per km long line per year in our calculations, the effects of applying the max/best value for production of seaweed becomes large and increases the SNPV to €2 526 thousand for the single firm scenario (an increase with 158%).

For the scale-up scenario, which is evaluated for a longer time period than the single-firm scenario, the *financial discount rate* has the second largest impact. Using 6% discount rate lowers the SNPV with 50% to €1.4 billion. However, the financial discount rate can be increased to 15.6% before the SNPV becomes negative. Using 2% discount rate increases the SNPV with 101% to €5.8 billion. *Production of seaweed* per km long line has the third largest impact (-9% and 123%), followed by recurrent and construction costs (± 4%), recreational loss (± 3%), and value of N and P uptake (± 1%).

If the best values (most favorable; 2 % discount rate) are used for all variables, the SNPV for the single-firm scenario is €4.9 million and €11.7 billion for the scale-up scenario. If the worst values (less favorable; 6 % discount rate) are used for all variables the SNPV for the single-firm scenario is minus €0.4 million and minus €138 million for the scale-up scenario. An "all best" or "all worst" outcome would however be unlikely and does not take correlation between variables into account. For example, labour and energy costs may decrease with lower harvest size, or increase with higher harvest size.

REFERENCES

Uncategorized References

1 Watson, L. & Dring, M. Business plan for the establishment of a seaweed hatchery and grow-out farm. *Irish Sea Fisheries Board: Dun Laoghaire, Ireland* (2011).

2 van den Burg, S. W. K., van Duijn, A. P., Bartelings, H., van Krimpen, M. M. & Poelman, M. The economic feasibility of seaweed production in the North Sea. *Aquaculture Economics & Management* **20**, 235-252, doi:10.1080/13657305.2016.1177859 (2016).

3 Camus, C., Infante, J. & Buschmann, A. H. Revisiting the economic profitability of giant kelp Macrocystis pyrifera (Ochrophyta) cultivation in Chile. *Aquaculture* **502**, 80-86, doi:https://doi.org/10.1016/j.aquaculture.2018.12.030 (2019).

4 Visch, W. *Doctoral Thesis: Sustainable Kelp Aquaculture in Sweden* Doctor of Philosophy thesis, Gothenburg University, (2019).

5 Riksbanken. *Average exchange rates for 2017*, <https://www.riksbank.se/sv/statistik/sok-rantor--valutakurser/arsgenomsnitt-valutakurser/?y=2017&m=12&s=Comma&f=y> (2018).

6 SCB. *Prices on electricity for industrial consumers 2007*, <https://www.scb.se/hitta-statistik/statistik-efter-amne/energi/prisutvecklingen-inom-energiomradet/energipriser-pa-naturgas-och-el/pong/tabell-och-diagram/genomsnittspriser-per-halvar-2007/priser-pa-el-for-industrikunder-2007/> (2018).

7 Watson, L. (2018).

8 SCB. *Average hourly pay, manual workers private sector (SLP) by pay element by industry SNI 2007, type of working time and sex. Year 2008 – 2018*, <http://www.statistikdatabasen.scb.se/pxweb/sv/ssd/START AM AM0103 AM0103A/SLP9a07/?rxid=86abd797-7854-4564-9150-c9b06ae3ab07 > (2018).

9 Verksamt. *Employee cost calculator.*, <https://www.verksamt.se/alla-e-tjanster/rakna-ut/rakna-ut-vad-en-anstalld-kostar> (2019).

10 SCB. *Average basic salary, monthly salary and women´s salary as a percentage of men´s salary by sector, occupation (SSYK 2012), sex and educational level (SUN) . Year 2014 – 2018*, <http://www.statistikdatabasen.scb.se/pxweb/sv/ssd/START AM AM0110 AM0110A/LonYrkeUtbildning4A/?rxid=04cb74d2-1dfd-427f-8c74-d1be7d42de65> (2018).

11 Le Bras, Q., Lesueur, M., Lucas, S. & GOUIN, S. Etude du marché français des algues alimentaires. Panorama de la distribution. Programme IDEALG Phase 2 - Programme IDEALG Phase 2. Les publications du Pôle halieutique AGROCAMPUS OUEST n°36. 42p (Agrocampus Ouest, 2015).

12 Tasende, M. G. & Peteiro, C. Explotación de las macroalgas marinas: Galicia como caso de estudio hacia una gestión sostenible de los recursos. *Revista Ambienta n* **111**, 116-132 (2015).

13 Organic Monitor. The European market for sea vegetables. (2014).

14 KosterAlg. *Our products*, <http://www.kosteralg.se/en/our-products/> (2019).

15 Ekonomifakta. *Productivity in Swedish industry*, <https://www.ekonomifakta.se/Fakta/Ekonomi/Produktion-och-

Investeringar/Produktivitetsutvecklingen-i-naringslivet/?graph=/23919/1,4/all/ > (2018).
16 Ekonomifakta. *Real wages in Sweden 1960-2018*, <https://www.ekonomifakta.se/Fakta/Arbetsmarknad/Loner/Loneutveckling-och-inflation/?graph=/20419/1/all/ > (2018).
17 Pechsiri, J. S. *et al.* Energy performance and greenhouse gas emissions of kelp cultivation for biogas and fertilizer recovery in Sweden. *Science of The Total Environment* **573**, 347-355, doi:http://dx.doi.org/10.1016/j.scitotenv.2016.07.220 (2016).
18 Ahlroth, S. *Valuation of environmental impacts and its use in environmental systems analysis tools*, KTH Royal Institute of Technology, (2009).
19 Kinell, G., Söderqvist, T. & Hasselström, L. *Monetära schablonvärden för miljöförändringar*. (Naturvårdsverket, 2009).
20 Noring, M. *Valuing ecosystem services - linking ecology and policy*, KTH Royal Institute of Technology, (2014).
21 Czajkowski, M. *et al.* Valuing the commons: An international study on the recreational benefits of the Baltic Sea. *Journal of environmental management* **156**, 209-217 (2015).
22 Ahtiainen, H. *et al.* Benefits of meeting nutrient reduction targets for the Baltic Sea–a contingent valuation study in the nine coastal states. *Journal of Environmental Economics and Policy* **3**, 278-305 (2014).
23 Helcom. in *HELCOM Extraordinary Ministerial Meeting.*
24 Söderqvist, T. *et al.* Effektkedjor och skadekostnader som underlag för revidering av ASEK-värden för luftföroreningar [Causal chains and damage costs as basis for revised ASEK values for air pollutants]. (Trafikverket, 2017).
25 Helcom. Updated Fifth Baltic Sea pollution load compilation. (2015).
26 SwAM. Marine tourism and recreation in Sweden. A study for the Economic and Social Analysis of the Initial Assessment of the Marine Strategy Framework Directive., (Swedish Agency for Marine and Water Management, Gothenburg, 2012).
27 Evans, D. in *Cost–Benefit Analysis and Incentives in Evaluation* (Edward Elgar Publishing, 2007).
28 Johansson, P.-O. & Kriström, B. *Cost-benefit analysis for project appraisal*. (Cambridge University Press, 2015).
